# Changes in the health status and health-related quality of life of community-dwelling older adults living alone: one-year follow-up from a cohort study

**DOI:** 10.3389/fpubh.2023.1278008

**Published:** 2023-12-21

**Authors:** Hana Ko, Belong Cho, Kyung-Choon Lim, Soong-Nang Jang, Sun Ju Chang, Yu Mi Yi, Hye Ryung Cho, So Im Ryu, Eun-Young Noh, Yeon-Hwan Park

**Affiliations:** ^1^College of Nursing, Gachon University, Incheon, Republic of Korea; ^2^Department of Family Medicine, College of Medicine, Seoul National University, Seoul, Republic of Korea; ^3^College of Nursing, Sungshin University, Seoul, Republic of Korea; ^4^Red Cross College of Nursing, Chung-Ang University, Seoul, Republic of Korea; ^5^College of Nursing, Seoul National University, Seoul, Republic of Korea; ^6^Institute of Nursing Science College of Nursing, Seoul National University, Seoul, Republic of Korea; ^7^Department of Nursing, Dong-Eui University, Busan, Republic of Korea; ^8^Department of Nursing, Changwon National University, Changwon-si, Republic of Korea; ^9^Department of Nursing, Konkuk University, Chungju, Republic of Korea

**Keywords:** health-related quality of life, living arrangements, health outcomes, aged, cohort

## Abstract

**Background:**

The percentage of older adults living alone is rapidly increasing, improving the health status and health-related quality of life (HRQoL) in this group is becoming a more significant public health issue. This study aimed to examine the changes in the HRQoL of older South Korean adults living alone and identify the factors that affect their HRQoL.

**Methods:**

A longitudinal study design was followed. Data were collected at baseline and 1-year follow-up. Participants consisting of 789 older adults living alone in S*City aged>65 years completed a cohort survey regarding health status and HRQoL from August 2018 to August 2019. Trained interviewers conducted face-to-face interviews with the participants using a validated questionnaire (physical health, mental health, social health, and HRQoL). Generalized estimating equations were used to assess the change in health status and the interaction effect of time and gender. Then, a stepwise multiple logistic regression analysis was performed to identify factors related to HRQoL.

**Results:**

Time differences were observed in the subjective evaluation of health status (SEH), IPAQ scores, frailty, nutritional status, and depression. Gender differences were observed in the SEH, IPAQ, frailty, loneliness, depression, and social support. The interaction between time and gender was observed in the IPAQ and HRQoL. At baseline, SEH, depressive symptoms, gender, frailty, and age were associated with HRQoL. After one year, HRQoL was associated with SEH, frailty, depressive symptoms, cost of living, suicidal thoughts, gender, social support, loneliness, and suicide attempts.

**Conclusion:**

Our results highlight that HRQoL is associated with physical health, mental health, and social support. Future detailed studies are needed to determine whether governments and communities can prevent depression, loneliness, and suicidal thoughts through psychological support and provide economic support to improve the quality of life of older adults living alone.

## 1 Introduction

In 2020, the global population aged ≥60 years reached more than 1 billion, accounting for 13.5% of the world’s population (1). Additionally, increasing life expectancy, decreasing mortality rates, changing family structure, and increasing the percentage of older adults living alone are reported annually. In 2021, the percentage of older adults living alone in South Korea was 35.1% (2). Older adults who are living alone have worse health status and health-related quality of life (HRQoL) (2), so they require more social functions and resources from social networks than other forms of living arrangements (3). Thus, because of this rapid increase in older adults living alone, improving the health status and HRQoL of this group is becoming a more significant public health issue (4, 5). The World Health Organization (WHO) promotes well-being through healthy aging, which maintains and develops functional abilities (1). In this context, social frailty is emphasized in gerontological studies (6). Therefore, social efforts are required to improve the quality of life, including the functional health of older adults living alone.

HRQoL is a broad and multidimensional concept that focuses on an individual’s perception of his or her position in life (7). It is affected by physical and mental capacities, functional abilities, and environmental aspects, such as social factors. A previous study has reported that HRQoL in older adults is associated with sociodemographic factors (8) physical health status (9), nutritional status (7, 10), and mental health (11). However, only a few studies have comprehensively investigated the association between health status and HRQoL among older adults living alone.

The WHO has proposed the International Classification of Functioning, Disability, and Health (ICF) model, which can explain the health, health status, and quality of life of various population groups living in the community and performing activities of daily life (12). The ICF model encompasses not only physical and mental functions but also social functions, such as activities and social participation; personal factors, such as an individual’s age, gender, and socioeconomic characteristics; and environmental factors related to personal health and life conditions (12). Therefore, it is evaluated as a biopsychosocial integrated model that explains health and quality of life (13). This ICF model is also used to predict the quality of life of older male adults living alone (14). In our previous study, we investigated how health status is associated with the quality of life of older adults living alone, focusing on relationships that differ by gender (15). However, a regression analysis with a cross-sectional design is limited because it can only provide assumptions regarding positive correlations between variables from a specific perspective (16). Therefore, a longitudinal follow-up study is needed to be confident of an association based on an integrated model that affects the quality of life of older adults living alone.

## 2 Study aim and hypotheses

This one-year follow-up study from a cohort (15) aimed to examine the changes in health status and HRQoL among older adults living alone, clarify the associations between health status and HRQoL to develop successful public health services, and contribute to a deeper understanding of this topic. We hypothesized that (1) health status would change after one year, (2) HRQoL would change after one year, and (3) the association between health status the HRQoL would change after one year.

## 3 Materials and methods

### 3.1 Study design and participants

The study population was derived from a prospective cohort of older adults living alone. The primary older adults (n = 1,023) were obtained from a previous cross-sectional study on community-dwelling older adults aged>65 years who were living alone between August and October 2018 in Siheung City, South Korea (15). In August 2019, 789 participants underwent a 1-year follow-up examination (follow-up rate: 77.1%) (Supplementary Table S1). The participants included in the study were at least 65 years of age, living alone in Siheung City, able to communicate orally, and provided written informed consent. After excluding participants who were living with others (*n* = 7), those who did not undergo follow-up (*n* = 226), and those who did not complete the questionnaire (*n* = 1), data from 789 older adults were analyzed.

### 3.2 Measurement

#### 3.2.1 General characteristics

The general characteristics were divided into the following: age [because of great diversity among different age groups in late life, they can be further categorized as follows: young-old [65–74 years], old [75–84 years], and oldest-old [≥85] (17)]; gender (men, women); marital status (not married, married, divorced, widowed); surviving child (yes or no); education level (illiteracy, elementary school, junior high school, high school, ≥ college), current religion status (yes or no), economic status (income per month, cost of living per month); and social activity. Social activity was assessed through participants’ self-reported responses to the question, “How often do you engage in social activity?” Participants could select among the following responses: “none/1–2 times per month/1–2 times per week/3–4 times or more per week.”

#### 3.2.2 Health status

The participants’ health status was assessed based on their physical, mental, and social health.

The subjective evaluation of health status (SEH) was assessed using the question, “What is your current general health?” “Compared to last year, how is your current health?” and “How is your health status compared with that of others of the same age?” Responses were provided on a 5-point Likert scale (1, very poor to 5, very good), and the score of each item was summed to obtain the total SEH score, with a higher score indicating better SEH.

The physical health of the participants was measured using the International Physical Activity Questionnaire – Short Form (IPAQ) (18). The IPAQ measures daily physical activities, including walking (low-intensity activity), moderate and vigorous-intensity activities, and sitting, in the past 7 days, and their duration (minutes) and frequency (days). The weekly total energy expenditure (MET) was calculated as the sum of weekly energy expenditure for each type of activity. The total MET is the sum of 3.3 × walking, 4.0 × moderate score, and 8.0 × vigorous score (MET minutes/ week).

Frailty was assessed using the Korean Frailty Index (19) and consisted of eight items: hospital admission, self-assessment of health status, polypharmacy, weight loss, depressive mood, incontinence, Time Up and Go test, and visual or auditory problems, using a yes/no response format. The total scores range from 0 to 8, with scores of 0–2, 3–4, and ≥ 5 indicating robust, pre-frailty, and frailty, respectively.

Nutritional status was measured using the Mini Nutritional Assessment (MNA^®^-SF), which comprises six items: food intake decline in the past 3 months, weight loss in the past 3 months, mobility, psychological stress or acute disease in the past 3 months, neuropsychological problems, and body mass index. The MNA^®^-SF was strongly correlated with the original total MNA score (r = 0.95), with a sensitivity of 97.9%, specificity of 100%, and diagnostic accuracy of 98.7% (20). The total score ranges from 0 to 14 points; nutritional status is categorized as normal (0–7 points), risk of malnutrition (8–11 points), and malnourished (12–14 points).

Loneliness was measured using the 20-item UCLA Loneliness Scale (21, 22) on a four-point Likert scale (1 = never, 2 = hardly ever, 3 = sometimes, 4 = often). Scores ranged from 20 to 80, with higher scores indicating a higher level of loneliness.

Depression was measured using the Korean version of the Short Form Geriatric Depression Scale (23), developed by Sheikh and Yesavage (24) which contains 15 items. Ten items indicated the presence of depression when answered positively, whereas the remaining five items indicated the presence of depression when answered negatively. The total score ranged from 0 to 15 and was categorized as follows: 0–5, normal; 6–9, mild depression; and 10–15, severe depression.

Suicidal thoughts and suicide attempts were measured using two questions (25). Participants were asked whether they had seriously thought about committing suicide and could answer a 0 (never done) to 10 (always) point visual analog scale (VAS). A higher VAS score for suicidal thoughts indicated more thoughts about suicide. Suicide attempts were measured by the question, “During the past 12 months, how many times did you actually attempt suicide?”

Social health of the participants was assessed using the Enhancing Recovery in Coronary Heart Disease Social Support Instrument (26). The Korean version consists of six items (27), based on perceived emotional, instrumental, informational, and appraisal support. The scale had a total score ranging from 6 to 12. Higher scores indicated greater social support.

#### 3.2.3 Health-related quality of life

HRQoL was measured using EuroQoL – 5 Dimensions (EQ-5D). It is a standardized instrument developed by the EuroQol Group, which can be used for a range of health conditions (28). The EQ-5D consists of five dimensions: mobility, self-care, usual activities, pain/discomfort, and anxiety/depression. The possible responses for each dimension included none, slight, moderate, severe, or extreme problems, and the responses were converted to quality of life scores using Korean value sets (29). The EQ-5D index score has been suggested using the time trade-off method, and the range is from −1, meaning the worst health, to 1, meaning perfect health. The higher the index, the higher the subject’s health-related quality of life.

EQ-VAS evaluates imaginable health status that ranges from 0 (worst condition) to 100 (best condition).

### 3.3 Data collection

All 69 assistants received preliminary training on the purpose and outline of the study and survey method before undergoing face-to-face surveys. A gerontological nurse practitioner was always present as participants completed the questionnaires to clarify any doubts they had. The participants were evaluated at follow-up, with a baseline assessment using the same measurement. They took approximately 40 min to complete the questionnaires and measurements and were given daily necessities as a small token of appreciation afterward.

### 3.4 Data analysis

Using SPSS version 23 (IBM Corp., Armonk, N.Y., United States), data on the longitudinal participants’ characteristics were analyzed through changes in descriptive analyses using the Wilcoxon signed rank test (for non-normally distributed variables) and chi-square test (for categorical variables). Generalized estimating equations were used to assess changes in health status and the interaction effect of time and gender. Then, we performed stepwise multiple logistic regression analysis to identify factors related to HRQoL. Before running the regression analyses, the independent variables were tested for multicollinearity using the tolerance value and variance inflation factor (VIF). If the tolerance value is <0.1 (30) and the VIF value is ≥10, multicollinearity is problematic (31). All comparisons were two-tailed, and statistical significance was set at a *p*-value <0.05.

## 4 Results

### 4.1 General characteristics

The general characteristics of the 789 participants at baseline and follow-up are shown in Table 1. There were significant differences in participants’ total monthly income (*p* < 0.001) and cost of living (*p* = 0.006) between baseline and follow-up.

**Table 1:** General characteristics of older adults living alone (*N* = 789).

Variable	Categories	Baseline	After 1 year	*p*-value
		*n* (%) or M ± SD	*n* (%) or M ± SD	
Age (year)	Young-old	300 (38.0)	270 (34.2)	0.050
	Old	444 (56.3)	452 (57.3)	
	Oldest-old	45 (5.7)	67 (8.5)	
Gender	Men	166 (21.0)	166 (21.0)	
	Women	623 (79.0)	623 (79.0)	
Marital status	Not married	22 (2.8)	22 (2.8)	
	Married	1 (0.1)	1 (0.1)	
	Divorced	149 (18.9)	149 (18.9)	
	Widowed	617 (78.2)	617 (78.2)	
Surviving child	Yes	723 (91.6)	716 (90.7)	0.594
	No	66 (8.4)	73 (9.3)	
Educational level	Illiteracy	313 (39.7)	313 (39.7)	
	Elementary school	237 (30.0)	237 (30.0)	
	Junior high school	110 (13.9)	110 (13.9)	
	High school	99 (12.6)	99 (12.6)	
	≥College	30 (3.8)	30 (3.8)	
Religion	Yes	515 (65.3)	510 (64.6)	0.833
	No	274 (34.7)	279 (35.4)	
Economic status	Incomes ($/month)	505.15 ± 347.41	546.21 ± 378.54	<0.001
	Cost of living($/month)	474.19 ± 357.54	494.31 ± 294.61	0.006
Social activity	None	216 (27.4)	207 (26.2)	0.652
	1–2 times/month	43 (5.4)	34 (4.3)	
	1–2 times/week	151 (19.1)	161 (20.4)	
	3–4 times or more/week	379 (48.0)	387 (49.0)	

### 4.2 Change in the health status and HRQoL of older adults living alone

**Figure 1:**
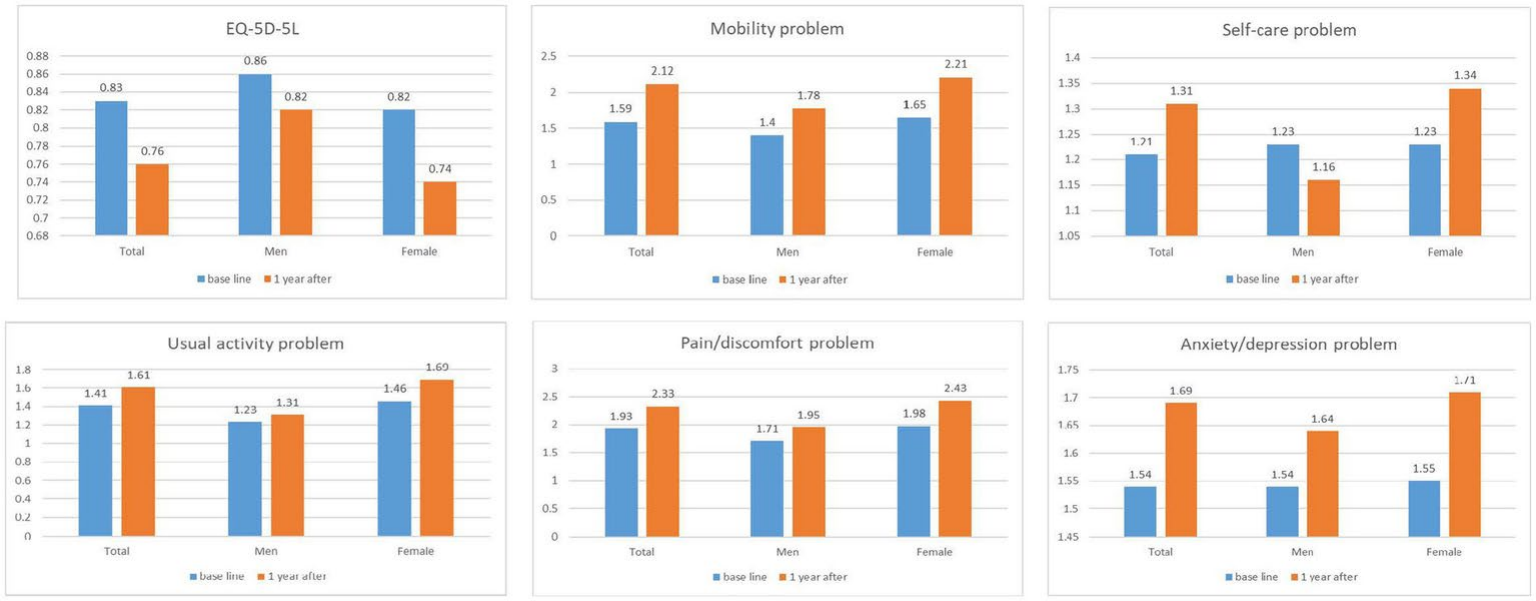
Health-related quality of life by time and gender.

The findings revealed more time and gender specificity in health status (Table 2); the physical health, mental health, social health, and quality of life of older adults living alone showed statistically significant differences in time and gender. Time differences were observed in SEH (*p* = 0.005), IPAQ scores (*p* = 0.042), frailty (*p* = 0.003), nutritional status (*p* < 0.001), and depression (*p* = 0.006). Gender differences were observed in SEH (*p* < 0.001), IPAQ (*p* < 0.001), frailty (*p* < 0.001), loneliness (*p* < 0.001), depression (*p* = 0.027), and social support (*p* < 0.001). However, the interaction between time and gender was only observed in the IPAQ score. The interaction effect of time and gender on EQ-5D-5L (*p* = 0.017), mobility (*p* = 0.024), usual activity (*p* = 0.028), and pain and discomfort (*p* = 0.032) were significant. EQ-VAS decreased after 1 year but was not statistically significant (*p* = 0.092). Figure 1 indicates a decrease in EQ-5D-5L and an increase in subcategory problems by time and gender, mobility, self-care, usual activity, pain and discomfort, and anxiety and depression problems.

**Table 2:** Changes in the health status and HRQoL of older adults living alone (*N* = 789).

Variable	Baseline			After 1 year			*p-value*		
	Total (*n* = 789)	Gender		Total (*n* = 789)	Gender				
		Men (*n* = 166)	Women (*n* = 623)		Men (*n* = 166)	Women (*n* = 623)	Time	Gender	Time × Gender
	*n* (%) or M ± SD	*n* (%) or M ± SD	*n* (%) or M ± SD	*n* (%) or M ± SD	*n* (%) or M ± SD	*n* (%) or M ± SD			
*Physical health*									
SEH	7.81± 2.68	8.64 ± 2.64	7.59 ± 2.65	8.09 ± 2.78	8.95 ± 2.86	7.86 ± 2.71	0.005	<0.001	0.874
IPAQ	1783.49 ± 3982.54	3390.23 ± 7152.61	1355.38 ± 2376.90	1668.55 ± 2444.14	2072.77 ± 2788.02	1560.85 ± 2334.72	0.042	<0.001	0.005
Frailty	2.88 ± 1.81	2.33 ± 175	3.02 ± 1.80	2.65 ± 1.83	2.11 ± 1.65	2.80 ± 1.85	0.003	<0.001	0.975
Normal	359 (45.5)	97 (58.4)	262 (42.1)	386 (48.9)	104 (62.7)	282 (45.3)	0.082	<0.001	0.807
Pre-frailty	272 (34.5)	47 (28.3)	225 (36.1)	267 (33.8)	44 (26.5)	223 (35.8)			
Frailty	158 (20.0)	22 (13.3)	136 (21.8)	136 (17.2)	18 (10.8)	118 (18.9)			
MNA^®^-SF	12.05 ± 2.09	12.24 ±1.96	12.00 ±2.12	11.77 ±2.17	11.77 ±2.25	11.77 ±2.16	<0.001	0.466	0.188
Normal	554 (70.2)	120 (72.3)	434 (69.7)	508 (64.4)	108 (65.1)	400 (64.2)	0.004	0.580	0.578
Risk of malnutrition	201 (25.5)	41 (24.7)	160 (25.7)	239 (30.3)	49 (29.5)	190 (30.5)			
Malnourished	34 (4.3)	5 (3.0)	29 (4.7)	42 (5.3)	9 (5.4)	33 (5.3)			
*Mental health*									
Loneliness	41.76 ± 13.06	45.05 ± 14.57	40.88 ± 12.50	41.79 ± 13.44	45.45 ± 13.72	40.82 ± 13.20	0.738	<0.001	0.644
Depression	6.38 ± 4.25	7.05 ± 4.45	6.19 ± 4.19	5.98 ± 4.30	6.54 ± 4.53	5.84 ± 4.23	0.006	0.027	0.614
Normal	370 (46.9)	69 (41.6)	301 (48.3)	405 (51.3)	77 (46.4)	328 (52.6)	0.063	0.059	0.832
Moderate	208 (26.4)	46 (27.7)	162 (26.0)	189 (24.0)	39 (23.5)	150 (24.1)			
Severe	211 (26.7)	51 (30.7)	160 (25.7)	195 (24.7)	50 (30.1)	145 (23.3)			
Suicide									
Thoughts	1.58 ± 2.84	1.94 ± 3.00	1.48 ± 2.79	1.50 ± 2.76	1.65 ± 2.85	1.46 ± 2.74	0.224	0.134	0.301
Attempt (yes)	68 (8.6)	18 (10.8)	50 (8.0)	75 (9.5)	15 (9.0)	60 (9.6)	0.994	0.593	0.228
*Social health*									
Social support	9.62 ± 1.98	8.84 ± 2.04	9.84 ± 1.91	9.66 ± 1.97	9.08 ± 2.07	9.81± 1.91	0.266	<0.001	0.160
*Health-related quality of life*									
EQ-5D-5L	0.83 ± 0.08	0.86 ± 0.10	0.82 ± 0.08	0.76 ± 0.20	0.82 ± 0.19	0.74 ± 0.21	<0.001	<0.001	0.017
Mobility	1.59 ± 0.53	1.40 ± 0.53	1.65 ± 0.52	2.12 ± 1.16	1.78± 1.10	2.21 ± 1.16	<0.001	<0.001	0.024
Self-care	1.21 ± 0.43	1.23 ± 0.35	1.23 ± 0.45	1.31 ± 0.72	1.16 ± 0.55	1.34 ± 0.75	0.007	<0.001	0.155
Usual activity	1.41 ± 0.53	1.23 ± 0.43	1.46 ± 0.54	1.61 ± 0.96	1.31 ± 0.70	1.69 ± 1.00	<0.001	<0.001	0.028
Pain/discomfort	1.93 ± 0.64	1.71 ± 0.68	1.98 ± 0.62	2.33 ± 1.21	1.95 ± 1.14	2.43 ± 1.21	<0.001	<0.001	0.032
Anxiety/ depression	1.54 ± 0.63	1.54 ± 0.69	1.55 ± 0.61	1.69 ± 1.01	1.64 ± 0.99	1.71 ± 1.02	0.001	0.513	0.457
EQ VAS	63.69 ± 20.86	65.86 ± 20.30	63.11 ± 20.98	62.47 ± 25.01	63.17 ± 23.02	62.29 ± 25.53	0.092	0.262	0.371

SEH, Subjective evaluation of health status; IPAQ, International Physical Activity Questionnaire; MNA^®^-SF, Mini-Nutritional Assessment – Short Form; EQ-5D, EuroQoL – 5 Dimensions scale.

### 4.3 Influencing factors in HRQoL

The results of the multiple regression analysis showed that the factors affecting the baseline HRQoL were age (ß = −0.085, *p* = 0.004), gender (ß = 0.172, *p* < 0.001), SEH (ß = 0.242, *p* < 0.001), frailty (ß = −0.162, *p* < 0.001), and depression (ß = −0.282, *p* < 0.001). When combined, these factors showed a 37.8% variability in baseline HRQoL. Conversely, after 1 year, participants’ HRQoL was significantly related to their gender (ß = 0.077, *p* = 0.008), cost of living (ß = 0.099, *p* < 0.006), SEH (ß = 0.291, *p* < 0.001), frailty (ß = −0.224, *p* < 0.001), depression (ß = −0.148, *p* < 0.001), loneliness (ß = 0.094, *p* = 0.011), suicidal thoughts (ß = −0.089, *p* = 0.006), social attempts (ß = −0.067, *p* = 0.016), and social support (ß = −0.110, *p* = 0.001). The combination of these variables showed 43.9% variability in follow-up HRQoL (Table 3). The tolerance value, which is a collinear statistic, was 0.614–0.902 for the baseline model and 0.433–0.920 for the follow-up model, all of them were ≥ 0.1. The VIF was 1.108–1.628 in the baseline model and 1.087–2.309 in the follow-up model, and all were below <10. Thus, multicollinearity was excluded.

**Table 3:** Influencing factors in health-related quality of life (*N* = 789).

Baseline						After 1 year					
Variables	*B*	*SE*	*ß*	*t*	*p*	Variables	*B*	*SE*	*ß*	*t*	*p*
(Constant)	0.914	0.037		24.829	<0.001	(Constant)	0.490	0.054		9.006	<0.001
Age	−0.001	0.000	−0.085	−2.852	0.004	Gender	0.039	0.015	0.077	2.645	0.008
Gender	0.035	0.006	0.172	5.734	<0.001	Cost of living	0.00007	0.000	0.099	3.533	<0.001
SEH	0.008	0.001	0.242	7.058	<0.001	SEH	0.021	0.003	0.291	8.305	<0.001
Frailty	−0.008	0.002	−0.162	−4.500	<0.001	Frailty	−0.025	0.004	−0.224	−6.294	<0.001
Depression	−0.006	0.001	−0.282	−8.270	<0.001	Depression	−0.007	0.002	−0.148	−3.636	<0.001
						Loneliness	0.001	0.001	0.094	2.564	0.011
						Suicidal thoughts	−0.007	0.002	−0.089	−2.747	0.006
						Suicidal attempts	−0.011	0.005	−0.067	−2.405	0.016
						Social support	−0.011	0.003	−0.110	−3.303	0.001
*R*^2^ = 0.378, adjusted *R*^2^ = 0.374, *F* = 95.264, *p* < 0.001						*R*^2^ = 0.439, adjusted *R*^2^ = 0.433, *F* = 67.776, *p* < 0.001					

SEH, Subjective evaluation of health status.

## 5 Discussion

This study examined the changes in the health status and HRQoL of South Korean older adults and changes in the factors affecting HRQoL to establish basic data for the development of health services that can improve the quality of life of older adults living alone. Most research variables of the general characteristics of older adults living alone were the same, except for economic status which changed statistically: income increased from US$505.15 to US$546.21, an increase of approximately 8.13%, and the cost of living increased 1 year after baseline. These results are related to the policies of the Korean Ministry of Health and Welfare. Thus, the sum of basic living benefits and old-age pensions for single-person households increased by 8.05% in 2019 from 2018 (32). Furthermore, the cost of living was found to be an associated variable for HRQoL. Therefore, this suggests that the government should continue to take into account the inflation rate and make efforts to guarantee a minimum cost of living for older adults living alone.

We also found that the MNA^®^-SF score significantly decreased over time. The nutritional status of older adults is related to not only the digestive function but also the sensory function. It is also related to economic status and worsens as aging progresses (33–35).

Interestingly, we found an interaction effect between differences in time and gender differences in HRQoL among older adults. In the present study, the EQ-5D-5L of older adults living alone decreased over the course of 1 year, and the change in women was greater than that of men. Moreover, this interaction between time and gender was found in mobility, usual activity, and pain/discomfort. These results are consistent with reports of a lower quality of life with increasing age for women than for men (15, 36, 37). Moreover, reducing and eliminating gender inequality is crucial for women and their ability to meet basic needs and improve their quality of life (1). Therefore, it is necessary to maintain quality of life by preventing the sudden deterioration of women’s health.

Regarding the factors influencing HRQoL in this study, SEH, depressive symptoms, gender, and frailty were significantly associated with both baseline and 1-year follow-up. This finding is in line with the results of previous studies (9, 10, 15, 38). Therefore, controlling depression and frailty by gender could lead to good HRQoL.

The suicide rate of older adult Korean individuals in 2019 was 46.6 per 100,000 deaths, a decrease of 2% compared with that noted in the previous year; however, Korea still ranks first among the member countries of the Organization for Economic Co-operation and Development (39). In terms of gender, the suicide rate for men and women decreased by 6.7 and 1.3%, respectively (39). The same results showed that the suicide rate for older adults living alone decreased after 1 year and in men but increased in women. Moreover, the primary reason for older Korean adults reporting suicide attempts was physical problems (42.2%), followed by mental and psychiatric problems (33.2%), and then financial concerns (12.3%) (39). A previous study on older adults with executive dysfunction showed that age ≥ 75 years, living alone, and low socioeconomic status were associated with suicidal ideation or attempts (37). In this context, suicidal thoughts can lead to a lower quality of life (11). In this study, frailty, depression, and cost of living were found to affect the quality of life along with suicide. Therefore, there is a need to improve the quality of life and suicide prevention through physical, mental, and economic interventions.

The WHO emphasizes community care and aging-friendly environments (1). Strengthening social support is emerging as an important policy because it prevents social weakness and improves the quality of life of older adults (40, 41). In a previous study, integrated social networks were associated with higher physical function and nutritional status at an 8-month follow-up (33). Moreover, Web-based message consumption had a more significant effect on reducing depressive symptoms in older adults over time than offline support networks (42). Thus, there is a need to develop social networks and support systems for older adults living alone, using information and communications technology to easily and frequently meet, integrate, and systematically approach physical, mental, and economic support.

### 5.1 Limitations

This study is the first attempt at a large-scale longitudinal study investigating the changes in health status and HRQoL of older adults living alone in an urban area of South Korea. This study has the following several limitations. First, the follow-up period was relatively short even if we attempted to conduct a cohort study. To improve the validity of the results, it is necessary to conduct more long-term observational studies of cohorts. Specifically, the health status and HRQoL of older adults living alone before the COVID-19 pandemic should be investigated. Longitudinal research is necessary to investigate the factors affecting changes in health status and quality of life of older adults in the current situation. Second, although efforts were made to approach them as comprehensively as possible, other potential confounding variables that might also affect the HRQoL of older adults living alone were not included in this study (such as years of widowhood and years to be living alone) and should be included in future studies. Thirdly, due to the convenience sampling method used in the study, the sample of this study was drawn from only one community in South Korea. This affected the representativeness of the questionnaire respondents and limited the generalisability of the conclusions. Finally, in order to examine the relationship between changes in health status and HRQoL, only participants who were followed up were included in this study. In order to reduce the dropout rate in the future, as those who dropped out were often the oldest-old (Supplementary Table S1), measures such as door-to-door surveys to follow up older adults should be considered.

## 6 Conclusion

We found changes in the health status and HRQoL of older adults living alone. There were differences in time and gender in physical health, mental health, and cognitive function. Furthermore, there were interaction effects between gender, time, physical activity, and HRQoL. We also found evidence that HRQoL is associated with physical health, mental health, and social support. To improve the quality of life of older adults living alone, it is necessary to provide economic support to prevent depression, loneliness, and suicidal thoughts through psychological support, and strengthen social support. Further research should establish a cohort in which the social frailty group, i.e., older adults living alone is investigated through continuous longitudinal observation. These findings have implications for public health efforts to provide gender-based community services and social and economic support and prevent frailty, depression, and suicide, which increase HRQoL in older adults living alone.

## Data availability statement

The raw data supporting the conclusions of this article will be made available by the authors, without undue reservation.

## Ethics statement

The studies involving humans were approved by the Institutional Review Board of Seoul National University Hospital. The studies were conducted in accordance with the local legislation and institutional requirements. The participants provided their written informed consent to participate in this study.

## Author contributions

HK: Conceptualization, Data curation, Investigation, Project administration, Validation, Writing – review & editing, Formal analysis, Methodology, Resources, Visualization, Writing – original draft. BC: Validation, Writing – review & editing, Supervision. K-CL: Supervision, Validation, Writing – review & editing. S-NJ: Supervision, Validation, Writing – review & editing. SC: Supervision, Validation, Writing – review & editing, Investigation. YY: Investigation, Writing – review & editing, Data curation, Methodology. HC: Investigation, Methodology, Writing – review & editing. SR: Investigation, Methodology, Writing – review & editing. E-YN: Investigation, Methodology, Writing – review & editing. Y-HP: Investigation, Writing – review & editing, Conceptualization, Data curation, Funding acquisition, Project administration, Supervision, Validation.
