# Correction: Changes in the health status and health-related quality of life of community-dwelling older adults living alone: one-year follow-up from a cohort study

**DOI:** 10.3389/fpubh.2026.1789508

**Published:** 2026-02-10

**Authors:** Hana Ko, Belong Cho, Kyung-Choon Lim, Soong-Nang Jang, Sun Ju Chang, Yu Mi Yi, Hye Ryung Cho, So Im Ryu, Eun-Young Noh, Yeon-Hwan Park

**Affiliations:** 1 College of Nursing, Gachon University, Incheon, Republic of Korea; 2 Department of Family Medicine, College of Medicine, Seoul National University, Seoul, Republic of Korea; 3 College of Nursing, Sungshin University, Seoul, Republic of Korea; 4 Red Cross College of Nursing, Chung-Ang University, Seoul, Republic of Korea; 5 College of Nursing, Seoul National University, Seoul, Republic of Korea; 6 Institute of Nursing Science College of Nursing, Seoul National University, Seoul, Republic of Korea; 7 Department of Nursing, Dong-Eui University, Busan, Republic of Korea; 8 Department of Nursing, Changwon National University, Changwon-si, Republic of Korea; 9 Department of Nursing, Konkuk University, Chungju, Republic of Korea

**Keywords:** health-related quality of life, living arrangements, health outcomes, aged, cohort

Affiliation 7 “Department of Nursing, Dong-Eui University, Busan, Republic of Korea” was erroneously given as “College of Nursing, Kyungnam College of Information and Technology, Busan, Republic of Korea”.

The original version of this article has been updated.

