# Supplementary material for: Changes in the health status and health-related quality of life of community-dwelling older adults living alone: one-year follow-up from a cohort study
Source: Front Public Health. 2023 Dec 21;11:1278008. doi: 10.3389/fpubh.2023.1278008 (PMC10772141; doi:10.3389/fpubh.2023.1278008)
Supplement: Supplementary file 1 [file Table_1.DOCX]

Table S1. Comparison of the baseline demographics of those analyzed and those excluded participants

| **Variable** | **Categories** | **Included (N=789)** | **Excluded (N=234)** | ***p-value*** |
| --- | --- | --- | --- | --- |
|  |  | n(%)or M$\pm$SD | n(%)or M$\pm$SD |  |
| **Age(year)** | Young-old | 300 (38.0) | 76 (32.5) | 0.006 |
|  | Old | 444 (56.3) | 121 (51.7) |  |
|  | Oldest-old | 45 (5.7) | 37 (15.8) |  |
| **Gender** | Men | 166 (21.0) | 60 (25.6) | 0.148 |
|  | Women | 623 (79.0) | 174 (74.4) |  |
| **Marital status** | Not married | 22 (2.8) | 6 (2.6) | 0.102 |
|  | Married | 1 (0.1) | 3 (1.3) |  |
|  | Divorced | 149 (18.9) | 44 (18.8) |  |
|  | Widowed | 617 (78.2) | 181 (77.4) |  |
| **Surviving child** | Yes | 723 (91.6) | 216 (92.3) | 0.742 |
|  | No | 66 (8.4) | 18 (7.7) |  |
| **Educational level** | Illiteracy | 313 (39.7) | 107 (45.7) | 0.204 |
|  | Elementary school | 237 (30.0) | 50 (21.4) |  |
|  | Junior high school | 110 (13.9) | 35 (15.0) |  |
|  | High school | 99 (12.6) | 34 (14.5) |  |
|  | ≥College | 30 (3.8) | 8 (3.4) |  |
| **Religion** | Yes | 515 (65.3) | 149 (63.7) | 0.653 |
|  | No | 274 (34.7) | 85 (36.3) |  |
| **Economic status** | incomes($/mon) | 505.15 $\pm$ 347.41 | 498.27 $\pm$ 396.98 | 0.796 |
|  | cost of living($/mon) | 474.19 $\pm$ 357.54 | 464.62 $\pm$368.92 | 0.811 |
| **Social activity** | None | 216 (27.4) | 73 (31.2) | 0.583 |
|  | 1~2times/month | 43 (5.4) | 12 (5.1) |  |
|  | 1~2times/week | 151 (19.1) | 38 (16.2) |  |
|  | 3~4 times or more/week | 379 (48.0) | 111 (47.4) |  |
